# Ethylene and Phloem Signals Are Involved in the Regulation of Responses to Fe and P Deficiencies in Roots of Strategy I Plants

**DOI:** 10.3389/fpls.2019.01237

**Published:** 2019-10-10

**Authors:** Carlos Lucena, Rafael Porras, María J. García, Esteban Alcántara, Rafael Pérez-Vicente, Ángel M. Zamarreño, Eva Bacaicoa, José M. García-Mina, Aaron P. Smith, Francisco J. Romera

**Affiliations:** ^1^Department of Botany, Ecology and Plant Physiology, Campus de Excelencia Internacional Agroalimentario CeiA3, Universidad de Córdoba, Córdoba, Spain; ^2^IFAPA, Centro Alameda del Obispo, Córdoba, Spain; ^3^Department of Agronomy, Campus de Excelencia Internacional Agroalimentario CeiA3, Universidad de Córdoba, Córdoba, Spain; ^4^Department of Environmental Biology, Faculty of Sciences, Universidad de Navarra, Pamplona (Navarra), Spain; ^5^Department of Biological Sciences, Louisiana State University, Baton Rouge, LA, United States

**Keywords:** ethylene, iron, phloem, phosphorus, signals

## Abstract

Iron (Fe) and phosphorus (P) are two essential mineral nutrients whose acquisition by plants presents important environmental and economic implications. Both elements are abundant in most soils but scarcely available to plants. To prevent Fe or P deficiency dicot plants initiate morphological and physiological responses in their roots aimed to specifically acquire these elements. The existence of common signals in Fe and P deficiency pathways suggests the signaling factors must act in conjunction with distinct nutrient-specific signals in order to confer tolerance to each deficiency. Previous works have shown the existence of cross talk between responses to Fe and P deficiency, but details of the associated signaling pathways remain unclear. Herein, the impact of foliar application of either P or Fe on P and Fe responses was studied in P- or Fe-deficient plants of *Arabidopsis thaliana*, including mutants exhibiting altered Fe or P homeostasis. Ferric reductase and acid phosphatase activities in roots were determined as well as the expression of genes related to P and Fe acquisition. The results obtained showed that Fe deficiency induces the expression of P acquisition genes and phosphatase activity, whereas P deficiency induces the expression of Fe acquisition genes and ferric reductase activity, although only transitorily. Importantly, these responses were reversed upon foliar application of either Fe or P on nutrient-starved plants. Taken together, the results reveal interactions between P- and Fe-related phloem signals originating in the shoots that likely interact with hormones in the roots to initiate adaptive mechanisms to tolerate deficiency of each nutrient.

## Introduction

Iron (Fe) and phosphorus (P) are two essential elements for plant growth (Marschner, 1995). Both elements are abundant in most soils but are present in chemical forms generally not available to plants. As a result, plants enhance acquisition of Fe and P through morphological and physiological responses in their roots. Deficiency of either nutrient initiates similar root responses, such as development of subapical root hairs or cluster roots and the acidification of the rhizosphere or the production of organic acids (Lucena et al., 2015; Song and Liu, 2015; Neumann, 2016; Stetter et al., 2017; Bhosale et al., 2018; Lucena et al., 2018). A defining feature of Strategy I plants is the need to reduce Fe(III), the most abundant form of Fe in soils, to Fe(II), prior to acquisition. In *Arabidopsis*, this Fe(III) reduction is catalyzed by a ferric reductase (EC 1.16.1.17) encoded by the *FRO2* gene, whereas Fe(II) uptake is mediated by a transporter encoded by the *IRT1* gene (Walker and Connolly, 2008; Ivanov et al., 2012). Both genes are up-regulated under Fe deficiency and are activated by specific bHLH transcription factors, which are also up regulated by Fe deficiency. These bHLH transcription factors include FIT, bHLH38, bHLH39, and others in *Arabidopsis* (Yuan et al., 2008; Ivanov et al., 2012; Brumbarova et al., 2015). Some of the physiological responses to phosphate (Pi) starvation include the up-regulation of Pi transporters (e.g. AtPht1;4, also named AtPT2) and of phosphatase activities (e.g. AtPAP17, also named AtACP5) (Zhang et al., 2014). del Pozo et al. (1999) purified and sequenced the N-terminal region of a Pi starvation induced acid phosphatase (encoded by the *AtPAP17* gene) from *Arabidopsis thaliana*. PAP17 shares the characteristics of type 5 acid phosphatases, a class of purple acid phosphatases (PAP) stimulated under low P availability. Recent studies have demonstrated that secretion of PAPs can facilitate utilization of organic P in the rhizosphere (Wang et al., 2009; Robinson et al., 2012; Mehra et al., 2017).

Studies in recent years have demonstrated an important role of MYB type transcription factors on the expression of genes that initiate responses to Fe or P deficiency. *AtMYB72* increases its transcription under Fe deficiency and is involved in some Fe deficiency responses (García et al., 2010). Most transcription factors that induce P deficiency responses belong to the MYB family (Müller et al., 2007). Of particular note, the *Arabidopsis* MYB transcription factors AtPHR1 and AtPHR2 (“Phosphate Starvation Response” 1 and 2 respectively), are crucial for P deficiency signaling (Rubio et al., 2001; Franco-Zorrilla et al., 2004; Todd et al., 2004; Bari et al., 2006; Mehra et al., 2017).

In previous works, it has been found that P deficiency induces the expression of Fe-related genes; that Fe deficiency induces the expression of P-related genes and that both deficiencies induce the expression of ethylene-related genes (reviewed in García et al., 2015; Lucena et al., 2015; Lucena et al., 2018). Taken together, these results suggest the existence of cross talk between Fe and P deficiency. The upregulation of Fe acquisition genes under P deficiency is generally associated to the increased accumulation of Fe in leaves (Wang Y. H. et al., 2002; Misson et al., 2005; Hirsch et al., 2006; Ward et al., 2008; Zheng et al., 2009; Abel, 2011; Perea-García et al., 2013; Wang et al., 2014, Lucena et al., 2018). On the other hand, P fertilization excess causes Fe chlorosis in calcareous soils (Romera et al., 1992; Sánchez-Rodriguez et al., 2013; Henry et al., 2017) and Fe deficiency affects P content in leaves (de Kock, 1955).

Either work in the nineties revealed links between ethylene and responses to deficiencies of Fe (Romera and Alcántara, 1994) and P (Borch et al., 1999). Although the regulation of the genes related to these responses is not totally understood, ethylene has since been implicated in the activation of both Fe-related genes (*AtFIT*, *AtFRO2*, and *AtIRT1*; Lucena et al., 2006; Lucena et al., 2015; Lucena et al., 2018; García et al., 2011; García et al., 2015) and P-related genes (*AtPht1;4* and *AtPAP17*; Lei et al., 2011; Roldán et al., 2013). In all cases, the activating effect of ethylene was dependent on the Fe or P status of the plants (García et al., 2011; Lei et al., 2011; Neumann, 2016; Liu et al., 2017), which suggests the involvement of signals other than ethylene in gene activation (García et al., 2013). García et al. (2013) showed that the foliar application of Fe blocked the expression of Fe-acquisition genes in the *Arabidopsis* wild-type cultivar Col-0 but not in the *Arabidopsisopt3-2* mutant, which suggests the existence of a Fe-related repressive signal moving in the phloem. This mutant harbors a T-DNA insertion in the *AtOPT3* promoter resulting in reduced *AtOPT3* expression (Stacey et al., 2008). Recently, *AtOPT3* has been implicated in the transport of Fe ions into the phloem (Zhai et al., 2014). In relation to ethylene, the *opt3* mutant produces more ACC in Fe-sufficient roots than the wild-type cultivar Col-0 (García et al., 2018). In agreement with this, it also presents constitutive activation of several ethylene-related genes in Fe-sufficient roots (García et al., 2018). These results suggest that a Fe-related repressive signal entering the phloem through OPT3 blocks ethylene synthesis in roots (**Figure 9**; García et al., 2018).

The *Arabidopsis pho2* mutant is impaired in the sensing of P-related phloem signals coming from leaves to roots and overaccumulates Pi in leaves under Pi-replete conditions (Bari et al., 2006; Chiou and Lin, 2011). Micrografting experiments revealed that a *pho2* root genotype is sufﬁcient to yield leaf Pi over-accumulation (Bari et al., 2006). *PHO2* encodes an ubiquitin-conjugating E2 enzyme, that targets proteins involved in P acquisition, like Pht1;4, and P loading into the xylem, like PHO1, for degradation (**Figure 9**; Chiou and Lin, 2011; Liu et al., 2012; Ham et al., 2018; Wang et al., 2018). Probably, PHO2 is also implicated in the regulation of PPAs protein stability (Wang and Liu, 2018). Under P deficiency, increased miRNA399, that participates in the cleavage of *PHO2* mRNA, enhances plant P acquisition and translocation by reducing the level of PHO2 protein (**Figure 9**; Chiou and Lin, 2011; Liu et al., 2012; Ham et al., 2018; Wang et al., 2018). miRNA399 is upregulated earlier in shoots than in roots and it has been proposed that its movement through the phloem acts as a systemic signal. In this sense, PHO2 is recognized as a systemic root sensor of shoot-derived P-related signals (Chiou and Lin, 2011).

The role of signals besides ethylene in the regulation of Fe and P acquisition genes is also supported by the fact that the *Arabidopsis* constitutive ethylene mutant *ctr1* does not present constitutive expression of either Fe-related genes (García et al., 2014) or P-related genes (Lei et al., 2011) when grown in complete nutrient solution. Among the non-ethylene signals involved in the regulation of Fe- or P-related genes have been proposed a Fe-peptide (García et al., 2013) and the miRNA399 described above (Pant et al., 2009; Kumar et al., 2017), both moving through the phloem. These signals could interact with ethylene and confer specificity to the responses to Fe or P deficiency, avoiding the induction of the specific responses when ethylene increases due to other nutrient deficiencies or stresses. Besides the specificity conferred by these non-ethylene signals, ethylene itself could confer specificity to the responses to Fe or P deficiency by acting through different signaling pathways in each case. For example, P deficiency did not induce the expression of P-related genes in the *Arabidopsis* ethylene insensitive mutants *ein2* and *etr1* (Lei et al., 2011), whereas Fe deficiency induced the expression of Fe-related genes in both mutants (Lucena et al., 2006; García et al., 2013). Taken together, the above results indicate that ethylene could regulate different nutrient responses by acting in conjunction with other signals and also by acting through different signaling pathways. More knowledge of the specific aspects of the regulation by ethylene of the responses to these deficiencies is necessary to understand the interactions between Fe and P. In this work we have further studied the existence of cross talks between the responses to Fe and P deficiency, taking into account the time course of both deficiencies and the existence of cross talks between Fe and P phloem signals. For this, we have studied Fe and P deficiency responses in *A. thaliana* Col-0, *opt3-2*, and *pho2* mutants, impaired in the transport of Fe through the phloem (*opt3-2*) or P homeostasis (*pho2*). The results obtained show that Fe deficiency induces the expression of P acquisition genes and phosphatase activity while P deficiency transitorily induces the expression of Fe acquisition genes and ferric reductase activity. Moreover, the foliar application of either Fe or P diminished the phosphatase activity and the expression of P-related genes in P deficient plants and the ferric reductase activity in Fe deficient plants. Taken together, the results show additional interactions between P and Fe signals originating in the shoots, and suggest that Fe- or P-related phloem signals can interact with ethylene for the regulation of the responses to their deficiencies.

## Materials and Methods

### Plant Material, Growth Conditions and Treatments

To analyze the effect of Fe or P deficiency and its interactions with Fe or P phloem signals on the induction of Fe or P deficiency responses and the expression of Fe or P acquisition genes, we used wild-type *Arabidopsis* [*A. thaliana* (L.) Heynh ecotype Columbia Col-0] and two *Arabidopsis* mutants [*opt3-2* (characterized by Stacey et al., 2008) and *pho2* (characterized by Delhaize and Randall, 1995)], which are affected in the transport of Fe (*opt3-2*) or sensing of P-related signals (*pho2*) in the phloem. Seeds of the *A. thaliana* mutants used in this study were obtained from Dr. Stacey (*opt3-2*) and Dr. Smith (coauthor of this research work; *pho2*). *Arabidopsis* plants were grown under controlled conditions as previously described (Lucena et al., 2006; Lucena et al., 2007). Briefly, seeds were germinated and, when appropriate, seedlings were transferred to individual containers of 70 ml volume with complete nutrient solution continuously aerated. The nutrient solution had the following composition: 2 mM Ca(NO_3_)_2_, 0.75 mM K_2_SO_4_, 0.5 mM KH_2_PO_4_, 0.65 mM MgSO_4_, 50 μM KCl, 10 μM H_3_BO_3_, 1 μM MnSO_4_, 0.5 μM CuSO_4_, 0.5 μM ZnSO_4_, 0.05 μM (NH_4_)_6_Mo_7_O_24_, and 20 μM Fe-EDDHA. When plants were 45-days-old, they were transferred from this complete nutrient solution to the different treatments. Nutrient solutions were renewed every week previously to the treatments but not during the treatments.

The treatments imposed were: Control: complete nutrient solution with 40 μM FeEDDHA and P; –Fe: nutrient solution without Fe for 2 days; –P: nutrient solution without P (0.5 mM KH_2_PO_4_ was not added to the nutrient solution, instead 0.5 mM KCl was added) for 7 days; –Fe–P: nutrient solution without Fe (the last 2 days) and without P for 7 days. The duration of the treatments was of 2 days for Fe deficiency and of 7 days for P deficiency because this is the time (in our experimental conditions of hydroponic culture system) for plants to exhibit maximal induction of their Fe or P acquisition genes. After these periods of time (2 days without Fe and 7 days without P) plants were remove for different assessments. Six replicates were used per each control or deficiency treatment.

In some treatments, the shoots were sprayed with 0.05% FeSO_4_ or 0.35% KH_2_PO_4_, both dissolved in deionized water, just 24 h before the end of the assay. A hand pressure sprayer was used for the foliar treatments.

### Acid Phosphatase Activity Determination

It was determined as previously described (Zakhleniuk et al., 2001). Briefly, roots of intact plants were placed in Petri dishes containing a solution with 5-bromo-4-chloro-3′-indolyphosphate p-toluidine salt (BCIP) 0.01% (w/v) for 4 h. Blue color of roots is higher with increased acid phosphatase activity. After 4 h, photographs of roots were taken with a stereoscopic microscope.

### Root Ferric Reductase Activity Determination

It was determined as previously described (Lucena et al., 2006; Lucena et al., 2007). Briefly, intact plants were placed in a Fe(III) reduction assay solution containing Fe(III)-EDTA and Ferrozine for 60 min. The ferric reductase activity was determined spectrophotometrically by measuring the absorbance (562 nm) of the Fe(II)-Ferrozine complex and using an extinction coefficient of 29,800 M^−1^cm^−1^. After the reduction assay, roots were excised and weighed, and the results were expressed on a root fresh weight basis. Finally, the roots were collected and kept at −80ºC for subsequent analysis of mRNA levels. Each experiment was repeated at least twice and representative results are presented. Data are given as means ± SE (n = 6).

### Analysis of Root ACC Content

The extraction, purification, and quantification of ACC (1-aminocyclopropane-1-carboxylic acid) was carried out using the method described by Mora et al. (2012). Briefly, ACC of roots was extracted with 20 μl of d_4_ACC [3 μg/ml in acetonitrile/acetic acid 0.2% (90/10)] and 3 ml of MeOH/H_2_O/HCOOH (15/4/1, v/v/v) at −20°C. Purification was carried out using a Strata C18-E cartridge (Ref 8B-S001-FBJ, Phenomenex, Torrance, CA, USA) preconditioned with 4 ml of methanol and 2 ml of MeOH/H_2_O/HCOOH (15/4/1, v/v/v). Finally the eluted fraction was centrifuged (10,000 rpm, 8 min) and injected in the LC/MS/MS systems, so ACC was quantified by HPLC linked to a 3200 QTRAP LC/MS/MS system (Applied Biosystems/MDS Sciex, Ontario, Canada), equipped with a turbo ion spray interface.

### qRT-PCR Analysis

Roots were ground to a fine powder with a mortar and pestle in liquid nitrogen. Total RNA was extracted using the Tri Reagent solution (Molecular Research Center, Inc., Cincinnati, OH) according to the manufacturer’s instructions. M-MLV reverse transcriptase (Promega, Madison, WI) was used to generate cDNA with 3 μg of total RNA from roots as template and random hexamers or oligo dT(20) as primers. Prior to cDNA synthesis, RNA was treated with DNAse to eliminate possible contamination by genomic DNA, being DNase inactivated later, adding 50 mM EDTA. Negative controls included all reaction components except M-MLV enzyme. One tenth of each RT reaction was used as PCR template.

The study of gene expression by qRT-PCR was performed using a qRT-PCR Bio-Rad (CFX connect), and the SYBR Green Bio-RAD PCR Master Mix, following the manufacturer’s instructions. *SAND1* and *YLS8* genes were used as reference genes to normalize the results of qRT-PCR. The Pfaffl method was used to calculate the relative expression levels. Primer pairs for *Arabidopsis* genes were designed as follows: (5´-3´) AtFRO2F (TGG TTG CCA CAT CTG CGT AT); AtFRO2R (TCG ATA TGG TGT GGC GAC TT); AtIRT1F (TGT CTC TTT TGC AAT CTG TCC A); AtIRT1R (AGG AGC TCC AAC ACC AAT CA); AtPAP17F (CCT CCA AGT ACG TTT CAT CGA TCC); AtPAP17R (CCG TGG CGG ACA TTA ACG AT).AtPht1;4F (CTT TTT CGG GTG GCT TGG TG); AtPht1;4R (AGC TTT TGG CTC ATG TCC GA).

### Statistical Analysis

All experiments were repeated at least twice and representative results are presented. A nonlinear regression test was applied to verify the relationship between number of hours under P deficiency conditions, genes expression and ferric reductase activity. The best regression model was chosen from many combinations of terms based, Akaike information criterion modified for small data sets, the model standard deviation and the coefficient of determination (*R^2^*). The analysis was performer by using analytical software 10.

The values of qRT-PCR represent the mean ± SE of three independent biological replicates. The values of other determinations (ferric reductase activity or ACC concentration) represent the mean ± SE of six replicates. Within each gene or genotype, different letters indicate significant differences (P < 0.05) among treatments using one-way analysis of variance (ANOVA) followed by a Duncan’s multiple range test. Dunnett’s test was also used when one or several mutants were compared with the WT for the +Fe treatment and when different treatments were compared with a control. In this latter case, ** indicate significant differences (P < 0.05).

## Results

### P Deficiency Induces the Enhancement of Ferric Reductase Activity and the Expression of Fe Acquisition Genes

**Figure 1:**
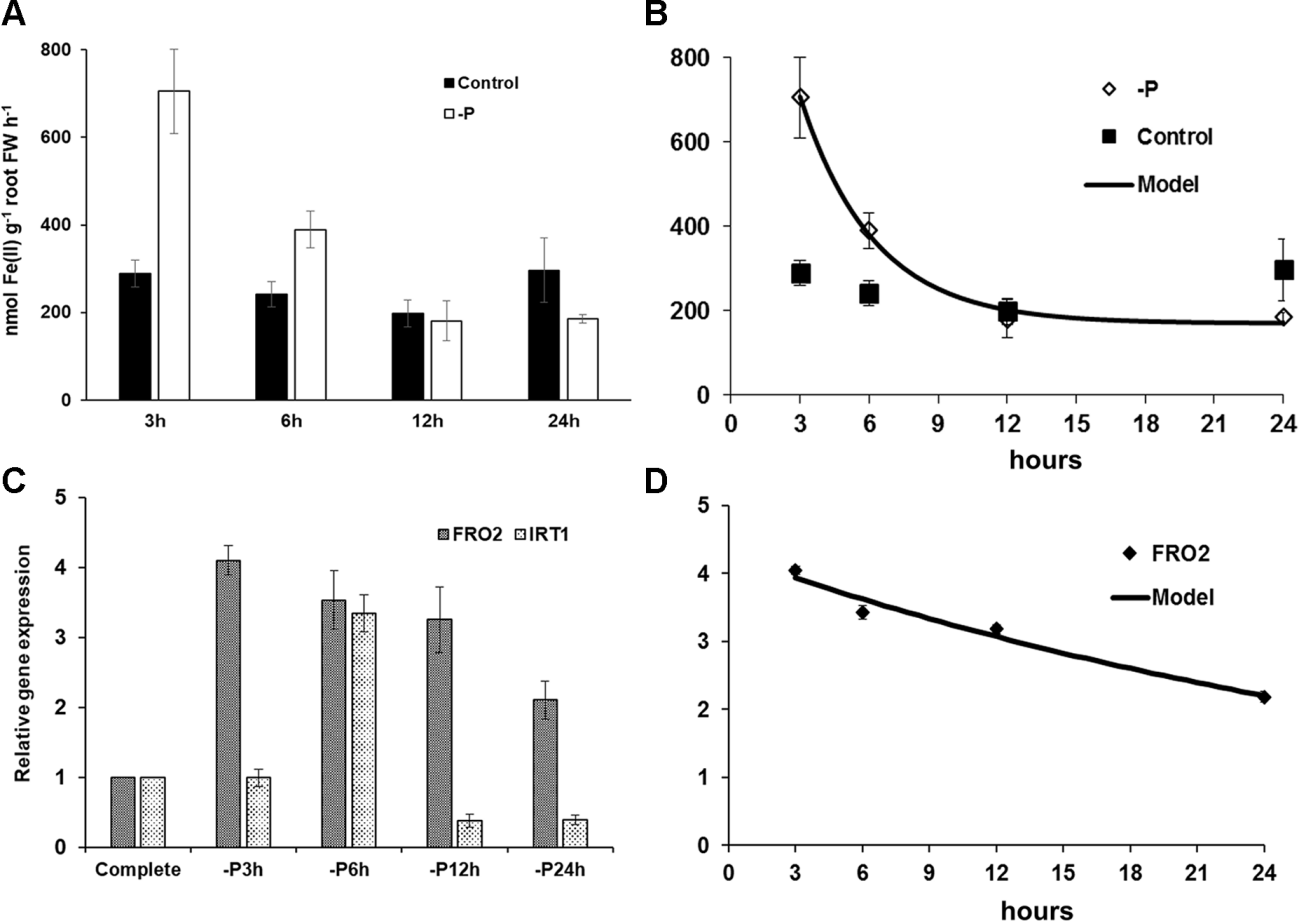
Effect of P deficiency on ferric reductase activity **(A)** and on *AtFRO2* and *AtIRT1* expression (both are Fe acquisition genes) **(C)** in *Arabidopsis* wild-type Columbia plants (Col-0). Plants were grown in a complete nutrient solution and some of them transferred to nutrient solution without P for 3, 6, 12, or 24 h. For ferric reductase activity **(A)**, data are given as means ± SE (n = 6). **(C)** qRT-PCR was performed using total RNA from roots as template and gene-specific primers to amplify partial cDNAs of *AtFRO2* and *AtIRT1*. *SAND1* and *YLS8* were used as reference genes to normalize the results obtained by qRT-PCR. Data represent the mean ± SE of three independent biological replicates and two technical replicates. A nonlinear regression test was applied over ferric reductase activity **(B)** R^2^ = 0.995; standard deviation = 27.33 and over *AtFRO2* expression **(D)** R^2^ = 0.966; standard deviation = 0.246.

The early effect of P deficiency on ferric reductase activity and on *AtFRO2* and *AtIRT1* expression (both are Fe acquisition genes) in *Arabidopsis* wild-type Columbia (Col-0) plants is shown in **Figure 1**. Just 3–6 h after transferring the plants to P deficiency conditions, both the ferric reductase activity and the Fe acquisition genes *AtFRO2* and *AtIRT1* were induced (**Figures 1A–D**). The induction observed progressively decreased after 6 h of deficiency.

### Fe Deficiency Induces Acid Phosphatase Activity and the Expression of P Acquisition Genes

**Figure 2:**
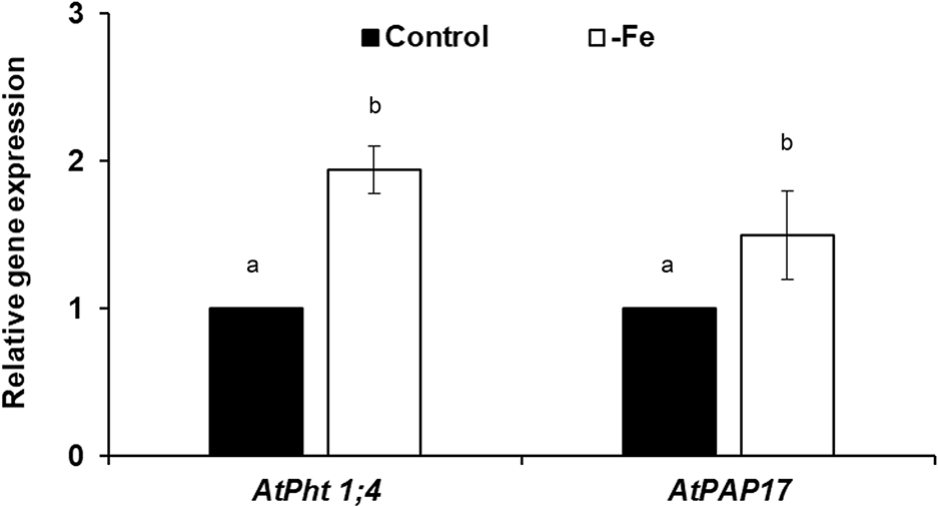
Effect of Fe deficiency on *AtPht1;4* and *AtPAP17* expression (both are P acquisition genes), in *Arabidopsis* wild-type Columbia plants (Col-0). Plants were grown in a complete nutrient solution and some of them transferred to nutrient solution without Fe for 2 days. Quantitative RT-PCR was performed using total RNA from roots as template and gene-specific primers to amplify partial cDNAs of *AtPht1;4* and *AtPAP17*. *SAND1* and *YLS8* were used as reference genes to normalize the results obtained by qRT-PCR. Relative expression was calculated in relation to Control. Data represent the mean ± SE of three independent biological replicates and two technical replicates. Within each gene, bars with different letters indicate significant differences (P < 0.05).

In relation to the P acquisition genes, both *AtPht1;4* and *AtPAP17* transcripts were detected in roots of Fe deficient plants compared to the control plants after 2 days of deficiency (**Figure 2**). Fe deficiency also induced phosphatase activity after 2 days (see **Figure 5A**).

### ACC Concentration is Higher in Both *opt3-2* and *pho2* Roots Than in Col-0 Roots

**Figure 3:**
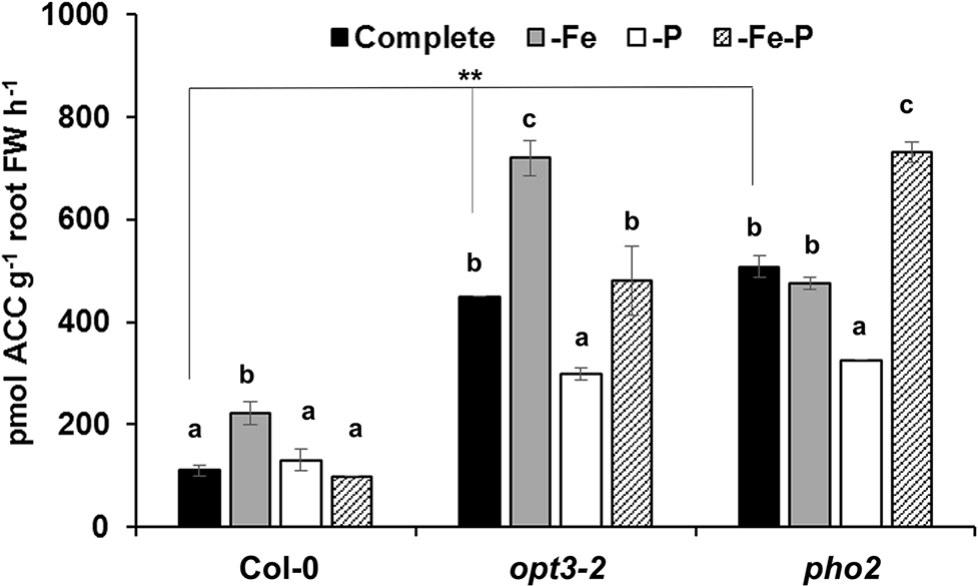
Effect of Fe-, P- or Fe- and P- deficiency on ACC production in *Arabidopsis* wild-type Col-0, *opt3-2* and *pho2* plants. Plants were grown in a complete nutrient solution and some of them transferred to nutrient solution without Fe, P, or Fe and P for 2 days. ACC production of roots was determined as previously described Mora et al. (2012). Data represent the mean ± SE (n = 6). Within each genotype, bars with different letters indicate significant differences (P < 0.05). Significant difference between the +Fe from Col-0, *opt3-2* and *pho2* is also indicated: **P < 0.05.

To look further into the relationship of ethylene with both deficiencies (see Introduction section; Nagarajan and Smith, 2012; Lucena et al., 2015), the effect of Fe, P, or Fe and P deficiency on ACC (an ethylene precursor) production was determined. As shown in **Figure 3**, both *opt3-2* and *pho2* mutant roots exhibited higher ACC concentrations than Col-0 roots in all treatments.

### Fe or P Foliar Applications Has a Differential Inhibitory Effect on Ferric Reductase Activity in Col-0 Plants or in *opt3-2* and *pho2* Mutant Plants

**Figure 4:**
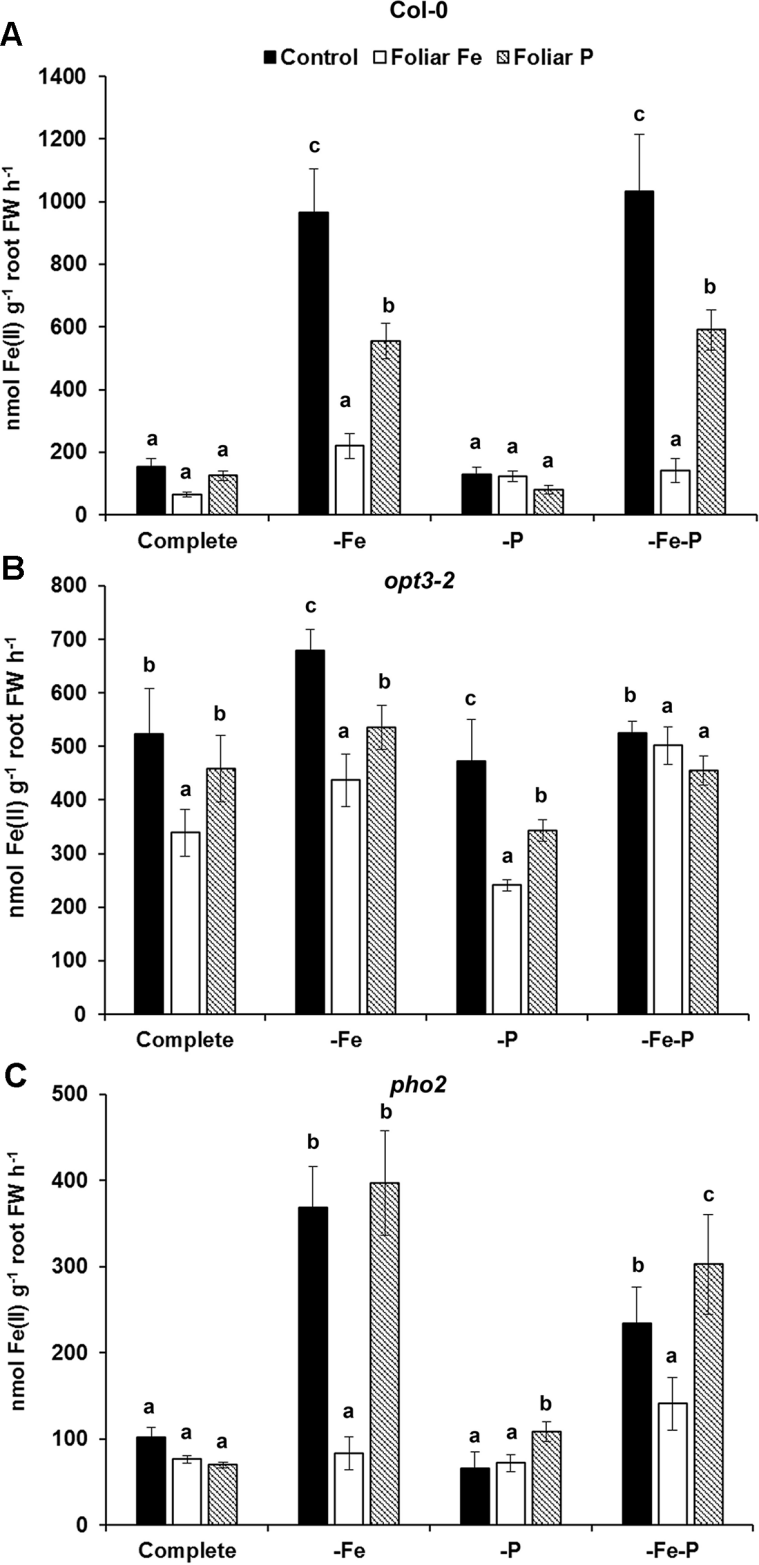
Effect of the foliar application of Fe or P on the ferric reductase activity of Fe-, P- or Fe- and P-deficient *Arabidopsis* wild-type Col-0 **(A)**, *opt3-2* **(B)** and *pho2* **(C)** plants. *opt3-2* and *pho2* are mutants affected in the transport of Fe (*opt3-2*) or sensing of P-related signals (*pho2*) in the phloem, respectively. Deficient treatment were: without Fe (–Fe) for 2 days; without P (–P), for 7 days; and without Fe and P (–Fe–P) (–Fe the last 2 days and –P for 7 days). Some of the plants were sprayed with 0.05% FeSO_4_ or 0.35% KH_2_PO_4_ just 24 h before the end of the assay. One day later, the ferric reductase activity was determined. Data are given as means ± SE (n = 6). Within each deficiency treatment, bars with different letters indicate significant differences (P < 0.05).

To study the cross talk between shoot Fe and P derived signals, we studied the effect of foliar applications of Fe or P on both Fe- and P-physiological responses. At first, the effects of the foliar applications of Fe or P on the ferric reductase activity was studied in Fe-, P-, and Fe–P-deficient plants of Col-0 and of the *opt3-2* and *pho2* mutants (**Figure 4**). These mutants are affected in the transport of Fe (*opt3-2*) or sensing of P-related signals (*pho2*) in the phloem. The induction of ferric reductase activity caused by -Fe (2 days) or –Fe–P (the last 2 days and 7 days, respectively) deficient treatments was drastically inhibited by the foliar application of Fe and, to a lesser extent, by the foliar application of P (**Figure 4A**). The inhibition caused by the foliar application of either Fe or P was less in the *opt3-2* mutant, which exhibits constitutive induction of Fe responses (**Figure 4B**). In the *pho2* mutant, the foliar application of Fe inhibited the induction of the ferric reductase activity caused by the –Fe or –Fe–P treatments while the foliar application of P did not (**Figure 4C**).

### Fe or P Foliar Applications Has a Differential Inhibitory Effect on Acid Phosphatase Activity and on the Expression of *AtPAP17* in Col-0 Plants or in *opt3-2* and *pho2* Mutant Plants

**Figure 5:**
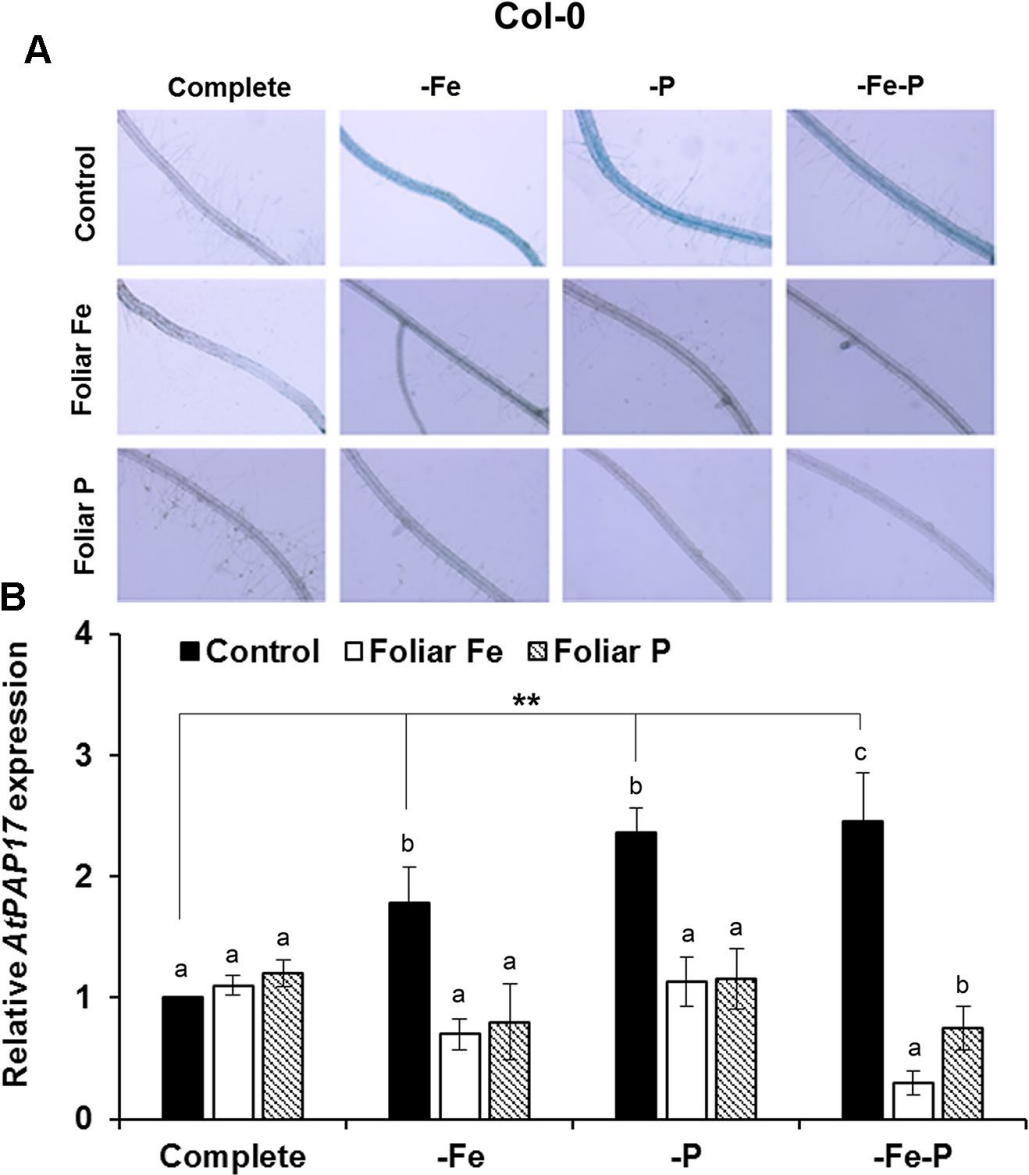
Effect of the foliar application of Fe or P on acid phosphatase activity **(A)** and on the expression of its encoding gene, *AtPAP17* **(B)**, in Fe-, P- or Fe- and P-deficient *Arabidopsis* wild-type Col-0 plants. For acid phosphatase activity determination, roots of intact plants were incubated in a solution containing a phosphated organic substrate (BCIP), for 4 h. Quantitative RT-PCR was performed using total RNA from roots as template and gene-specific primer to amplify partial cDNAs of *AtPAP17*. *SAND1* and *YLS8* were used as reference genes to normalize the results obtained by qRT-PCR. Data represent the mean ± SE of three independent biological replicates and two technical replicates. Within each deficiency treatment, bars with different letters indicate significant differences (P < 0.05). Significant difference between the control treatments is also indicated: **P < 0.05.

**Figure 6:**
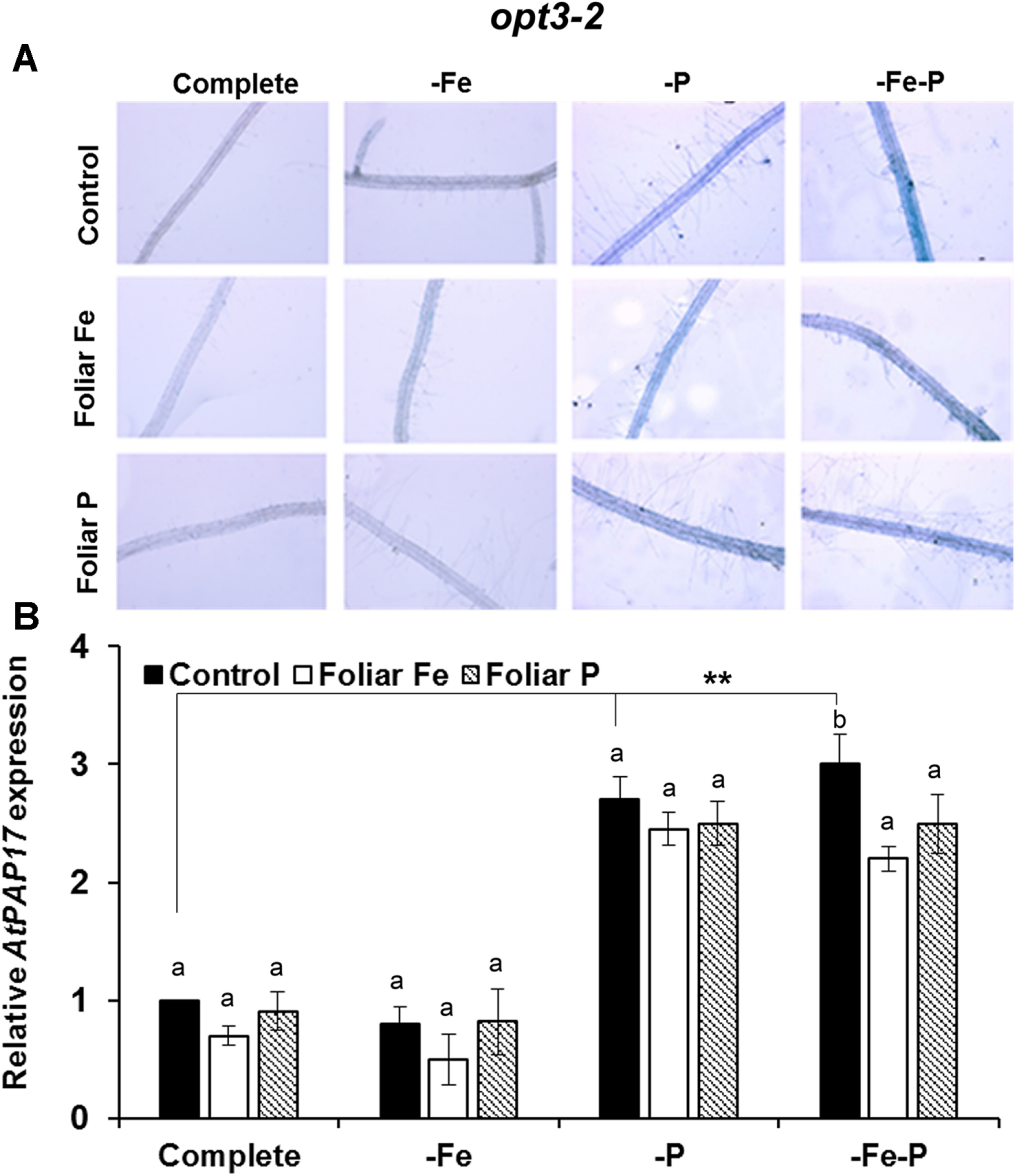
Effect of the foliar application of Fe or P on acid phosphatase activity **(A)** and on the expression of its encoding gene, *AtPAP17* **(B)**, in Fe-, P- or Fe and P-deficient *Arabidopsisopt3-2* mutant plants. This mutant is impaired in the phloematic transport of Fe. For acid phosphatase activity determination, roots of intact plants were incubated in a solution containing a phosphated organic substrate (BCIP), for 4 h. Quantitative RT-PCR was performed using total RNA from roots as template and gene-specific primer to amplify partial cDNAs of *AtPAP17*. *SAND1* and *YLS8* were used as reference genes to normalize the results obtained by qRT-PCR. Data represent the mean ± SE of three independent biological replicates and two technical replicates. Within each deficiency treatment, bars with different letters indicate significant differences (P < 0.05). Significant difference between the control treatments is also indicated: **P < 0.05.

**Figure 7:**
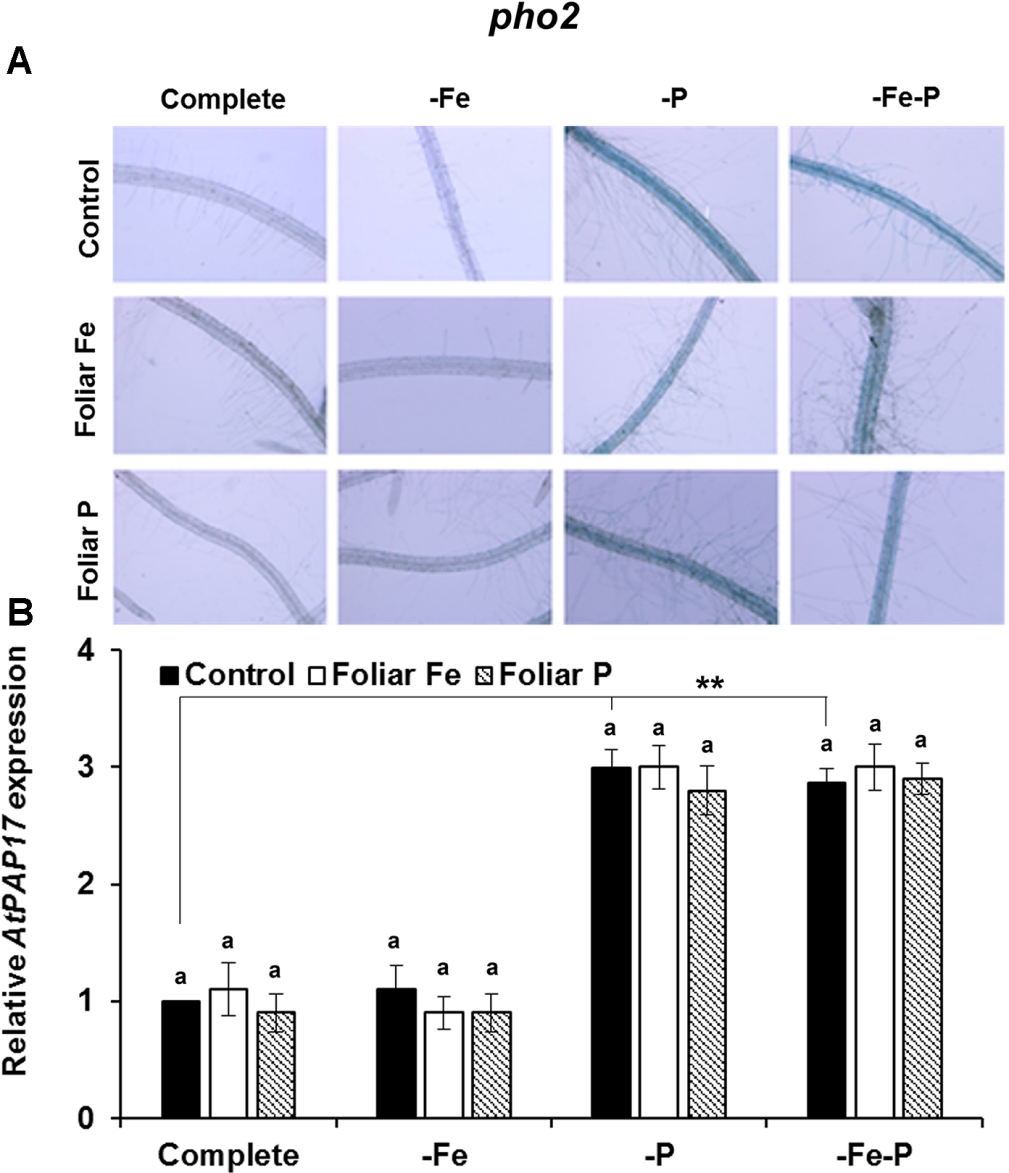
Effect of the foliar application of Fe or P on acid phosphatase activity **(A)** and on the expression of its encoding gene, *AtPAP17* **(B)**, in Fe-, P- or Fe- and P-deficient *Arabidopsispho2* mutant plants. This mutant is affected on sensing of P-related signals (*pho2*) in the phloem. For acid phosphatase activity determination, roots of intact plants were incubated in a solution containing a phosphated organic substrate (BCIP), for 4 h. Quantitative RT-PCR was performed using total RNA from roots as template and gene-specific primer to amplify partial cDNAs of *AtPAP17*. *SAND1* and *YLS8* were used as reference genes to normalize the results obtained by qRT-PCR. Data represent the mean ± SE of three independent biological replicates and two technical replicates. Within each deficiency treatment, bars with different letters indicate significant differences (P < 0.05). Significant difference between the control treatments is also indicated: **P < 0.05.

The effects of the foliar application of Fe or P on the acid phosphatase activity and on the expression of *AtPAP17* was studied in Fe, P, and Fe–P deficient plants of Col-0 (**Figure 5**) and of the *opt3-2* (**Figure 6**) and *pho2* mutants (**Figure 7**). All deficiency treatments applied caused induction of the phosphatase activity (**Figure 5A**) and *AtPAP17* expression (**Figure 5B**) in Col-0 plants. Just 2 and 7 days under Fe or P deficiency, respectively, were enough to induce both the phosphatase activity and *AtPAP17* expression (**Figures 5A, B**). These inductions were strongly inhibited by the foliar application of either Fe or P (**Figures 5A, B**). The induction of both phosphatase activity and *AtPAP17* expression in *opt3-2* and *pho2* was detected in –P and –Fe–P treatments, but not –Fe treatment (**Figures 6** and **7**). In contrast to Col-0, the inhibitory effect of the foliar application of Fe or P on the induction of both phosphatase activity and *AtPAP17* expression did not occur in either mutant (**Figures 6A, B** and **7A, B**). It could appreciate a slightly inhibitory effect of the foliar application of both Fe and P, only in –Fe–P treatment of *opt3-2* mutant plants (**Figure 6B**). In general, phosphatase activity and *AtPAP17* expression levels were strongly correlated (**Figures 5A, B**, **6A, B**, and **7A, B**).

### *opt3-2* Roots Present Constitutive Activation of Fe Acquisition Genes Under Complete Nutrient Solution but Not of P Acquisition Genes

**Figure 8:**
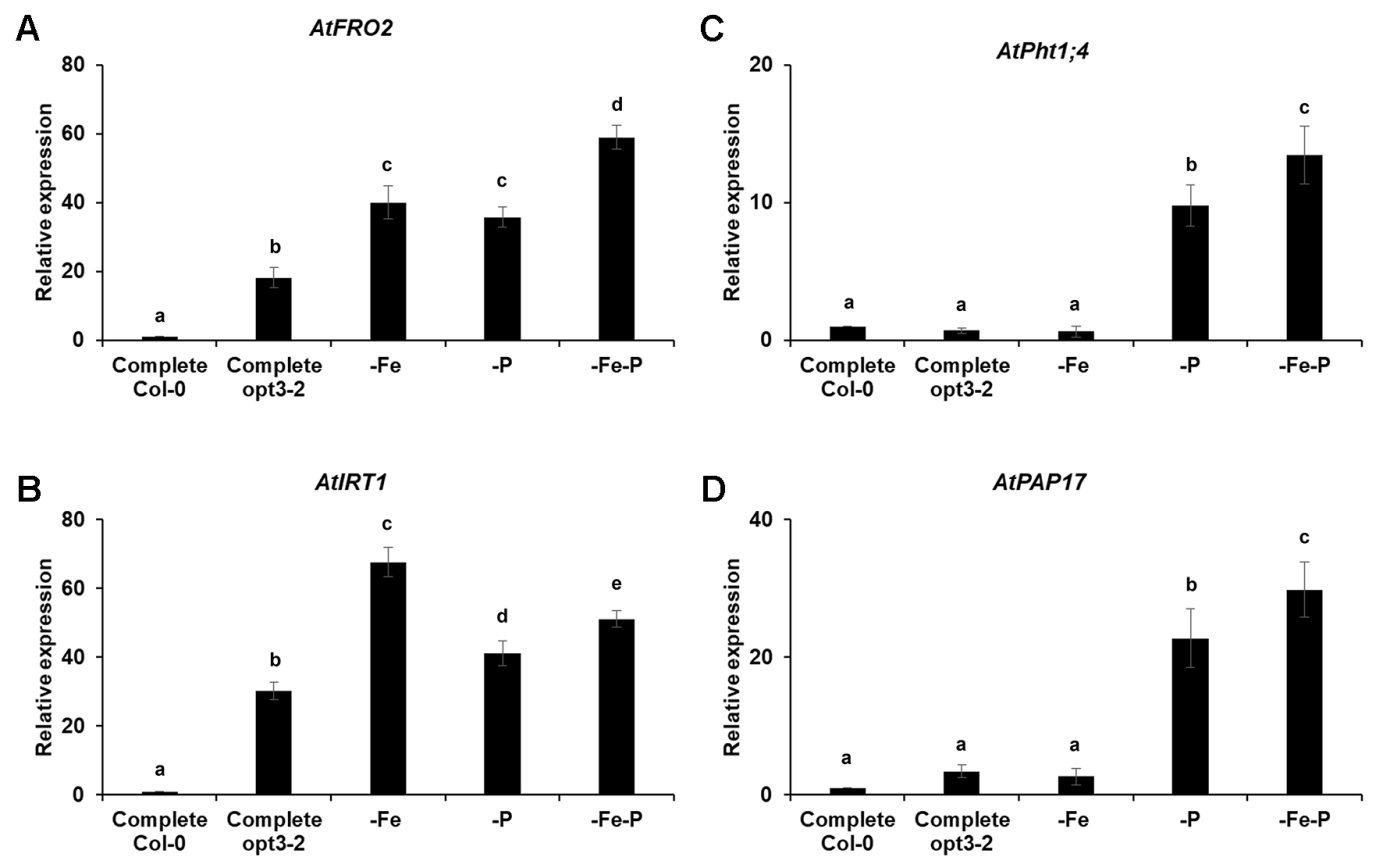
Effect of Fe-, P- or Fe- and P- deficiency on P acquisition gene expression **(C** and **D)** and Fe acquisition gene expression **(A** and **B)** in *Arabidopsisopt3-2* mutant plants. Plants were grown in a complete nutrient solution and some of them transferred to nutrient solution without Fe, P, or Fe and P for 2 days. Quantitative RT-PCR was performed using total RNA from roots as template and gene-specific primers to amplify partial cDNAs of *AtPht1;4*,*AtPAP17, AtFRO2* and *AtIRT1*. *SAND1* and *YLS8* were used as reference genes to normalize the results obtained by qRT-PCR. Data represent the mean ± SE of three independent biological replicates and two technical replicates. Within each gene, bars with different letters indicate significant differences (P < 0.05).

The results presented above suggest the existence of cross talk between Fe and P deficiency responses. However, this does not exclude the existence of specific signals that confer specificity to each deficiency. To analyze this possibility, we determined the expression of both Fe and P acquisition genes in the *opt3-2* mutant, which presents constitutive expression of Fe acquisition genes, even when grown under Fe sufficient conditions (Stacey et al., 2008; García et al., 2013). As shown in **Figures 8A, B**, this mutant exhibits almost constitutive activation of Fe acquisition genes in the different treatments. However, the P acquisition genes are not constitutively activated. They are only induced under P or Fe and P deficiency conditions (**Figures 8C, D**).

## Discussion

Previous works have shown the existence of cross talk between the responses to Fe and P deficiency in such a way that P deficiency induces the expression of Fe responses and Fe deficiency induces the expression of P responses. Those works have preferentially analyzed the expression of Fe- and P-related genes and Fe and P content in leaves (de Kock, 1955; Wang et al., 2002; Misson et al., 2005; Hirsch et al., 2006; Ward et al., 2008; Zheng et al., 2009; Abel, 2011; Perea-García et al., 2013; Wang et al., 2014). However, no studies have investigated the effect of each deficiency on some activities related to the other deficiency, such as ferric reductase activity (encoded by the *AtFRO2* gene in *Arabidopsis*) or phosphatase activity (encoded by the *AtPAP17* gene and other related genes in *Arabidopsis*). We think that, besides gene expression, it is important to determine these activities to better understand the cross talk between Fe and P deficiency because of the possible effects of post-transcriptional regulation. Additionally, we consider it important to study the time course of each deficiency on the induction of genes related to the other deficiency and the possible cross talk between shoot derived signals associated with each deficiency.

In this work, similar to previous works (see above), the results obtained show that P deficiency can induce the expression of some Fe acquisition genes, like *AtFRO2* and *AtIRT1* (**Figures 1C, D**; Wang et al., 2002; Misson et al., 2005; Wang et al., 2014), while Fe deficiency can induce the expression of some P acquisition genes, like *AtPht1;4* and *AtPAP17* (**Figure 2**). These results match with others obtained by microarray analysis showing an early transcriptional response of both *AtPht1;4* and *AtPAP17* to Fe deficiency (1–3 days growing without Fe) in roots of *Arabidopsis* plants (Thimm et al., 2001). The induction of the Fe acquisition genes by P deficiency occurred early, after 3 h (*AtFRO2*) and 6 h (*AtIRT1*) of the deficiency treatment, and then the induction tended to disappear (**Figures 1C, D**), which suggests the presence of specific Fe inhibitory signals. Moreover, the ferric reductase activity was induced only transitorily (**Figures 1A, B**) and decreased faster than the expression of the *AtFRO2* gene (**Figures 1C, D**), suggesting the possible existence of a post-transcriptional regulation by Fe (Connolly et al., 2003). In relation to Fe deficiency, it induced the expression of P acquisition genes and phosphatase activity after 2 days of deficiency (**Figures 2** and **5**), even before the induction caused by P deficiency itself (**Figure 5**). The induction of the phosphatase activity by Fe deficiency (**Figure 5A**) was not as transitory as the induction of ferric reductase by P deficiency (**Figure 1A**). This result is somewhat surprising since the induction of phosphatase activity is a response traditionally associated with P deficiency (Bari et al., 2006; Lei et al., 2011). The possible role of phosphatase activity on Fe nutrition is not known and warrants further investigation.

The existence of cross talk between the responses to Fe and P deficiency could be associated with common signals in the activation of both nutrient deficiency responses, such as ethylene (Nagarajan and Smith, 2012; García et al., 2015; Lucena et al., 2015; Song and Liu, 2015; Neumann, 2016; Lucena et al., 2018), nitric oxide (NO; Graziano and Lamattina, 2007; Meng et al., 2012) and auxin (Chen et al., 2010; Miura et al., 2011; Bhosale et al., 2018). In relation to ethylene, the results presented in **Figure 3** show that *opt3-2* and *pho2*, which exhibit constitutive Fe or P responses, respectively, have greater ACC content in roots than Col-0. Since both mutants are affected in the transport of Fe (*opt3-2*) or in the sensing of P-related signals (*pho2*) in the phloem (Bari et al., 2006; García et al., 2013), their higher ACC accumulation suggests that phloem-Fe signals and phloem-P signals can inhibit directly, or indirectly, ACC synthesis and, presumably, ethylene synthesis. These phloem signals should come from the shoots, as previously suggested (Buhtz et al., 2010; García et al., 2013).

To study the possible cross talk of Fe and P shoot-derived signals with the responses to both deficiencies, we sprayed leaves of Col-0, *opt3-2* and *pho2* plants with Fe or P, and analyzed their effects in roots. The foliar application of Fe inhibited the ferric reductase activity in Col-0 (**Figure 4A**) and in the *pho2* mutant (**Figure 4C**) while it had less effect in the *opt3-2* mutant (**Figure 4B**). This latter effect agrees with previous results (García et al., 2013). Curiously, the foliar application of P also inhibited the ferric reductase activity in Col-0 plants, less than the Fe application (**Figure 4A**), but had no effect on *pho2* (**Figure 4C**) and hardly in *opt3-2* (**Figure 4B**). These results suggest that there are Fe and P shoot-derived signals moving through the phloem that are inhibitory and that both Fe and P signals are interrelated. This interrelation is further supported when analysing phosphatase activity and the expression of one of its encoding genes, *AtPAP17*. As shown in **Figure 5**, the foliar application of either Fe or P drastically inhibited the phosphatase activity and *AtPAP17* expression in Col-0. However, neither Fe nor P application appreciably inhibited these P responses in either mutant (**Figures 6** and **7**).

Despite the existence of common signals involved in the activation of responses to both nutrient deficiencies, like ethylene (**Figure 3**), and the cross talk between shoot-derived signals (see previous paragraph), some results presented in this work suggest the existence of specific signals that block the activation of the responses to one deficiency when the deficiency is caused by the other element. For example, the expression of Fe acquisition genes is almost constitutively activated in the *opt3-2* mutant (**Figures 8A, B**) while the expression of P acquisition genes is not (**Figures 8C, D**). This suggests that the absence of the phloem-Fe signal related to OPT3 depresses the expression of Fe acquisition genes (García et al., 2013) but not that of P acquisition genes, probably because there are specific P signals that block it.

**Figure 9:**
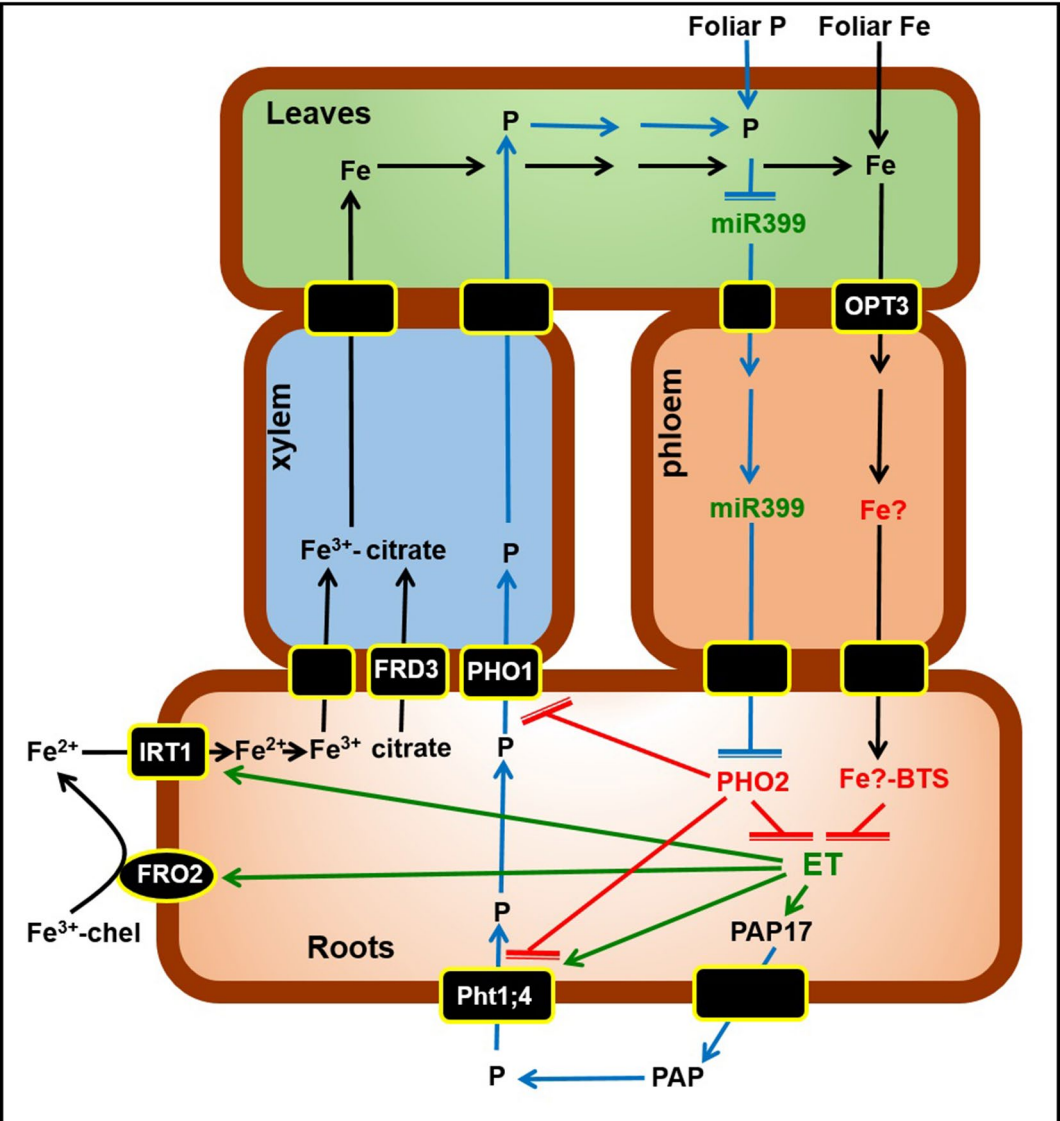
Working Model to explain the role of ethylene and P-related and Fe-related phloem signals on the regulation of Fe and P acquisition genes. Once inside roots, Fe (black arrows) is translocated to leaves through the xylem, bound to citrate (provided by the FRD3 transporter). In shoots, some Fe can enter the phloem through the OPT3 transporter, and moves back to roots probably bound to a chelating agent (Fe)?. In roots, this Fe? can be sensed by the Brutus protein (BTS) that blocks the expression of the Fe acquisition genes *FRO2* and *IRT1*, probably because inhibits the synthesis of ethylene (ET), which has been involved in their upregulation. P (blue arrows) is absorbed through P transporters, like Pht1;4, and then loaded into the xylem through transporters like PHO1. Under P deficiency, miR399 in shoots increases and moves through the phloem to roots where suppresses PHO2. PHO2 participates in the degradation of Pht1;4 and PHO1, and probably inhibits ethylene synthesis. Consequently, under P deficiency, the suppression of PHO2 by miR399 can permit the stabilization of Pht1;4 and PHO1, and the synthesis of ethylene, which has been involved in the upregulation of *Pht1;4* and *PAP17*, encoding a phosphatase (PAP). In green are components whose expression, activity and/or content is known to increase under Fe or P deficiency while in red are components whose expression, activity and/or content is known to increase under Fe or P sufficiency. For more details, see text. (→: promotion; 〒: inhibition). Working model based on Chiou and Lin (2011) and García et al. (2018).

According to the results obtained in this study we propose a Working Model to explain the role of ethylene and P-related and Fe-related phloem signals on the regulation of Fe and P acquisition genes (**Figure 9**). Once inside roots, Fe (black arrows) is translocated to leaves through the xylem, bound to citrate (provided by the FRD3 transporter). In shoots, some Fe can enter the phloem through the OPT3 transporter, and moves back to roots probably bound to a chelating agent (Fe)?. In roots, this Fe? can be sensed by the Brutus protein (BTS) that blocks the expression of the Fe acquisition genes *FRO2* and *IRT1*, probably because it inhibits the synthesis of ethylene (ET), which has been involved in their upregulation. P (blue arrows) is absorbed through P transporters, like Pht1;4, and then loaded into the xylem through transporters like PHO1. Under P deficiency, miR399 in shoots increases and moves through the phloem to roots where it suppresses PHO2. PHO2 participates in the degradation of Pht1;4 and PHO1, and probably inhibits ethylene synthesis. Consequently, under P deficiency, the suppression of PHO2 by miR399 can permit the stabilization of Pht1;4 and PHO1, and the synthesis of ethylene, which has been involved in the upregulation of *Pht1;4* and *PAP17*, encoding a phosphatase (PAP).

In conclusion, the results obtained in this work further support the cross talk between Fe and P deficiency responses since Fe deficiency induces the expression of P acquisition genes and the phosphatase activity, and P deficiency induces the expression of Fe acquisition genes and the ferric reductase activity. In most cases, like the induction of Fe acquisition genes and ferric reductase activity by P deficiency, this occurs very transitorily, probably due to the existence of Fe-related inhibitory signals and because of the post-transcriptional regulation of FRO2 by Fe. The cross talk between both deficiencies could be related to the existence of common signals, like ethylene, implicated in the activation of their responses. Besides ethylene, the results obtained with the foliar application of Fe or P show additional interactions between P and Fe inhibitory signals coming from the shoots, and suggest that Fe- or P-related phloem signals could interact with ethylene in the regulation of the responses to their deficiencies.

## Author Contributions

CL and FR designed the experiments after discussions with JG-M and MG; RP and MG conducted the laboratory work; ÁZ and EB determined ACC; AS, EA, RP-V, and CL wrote the manuscript.

## Funding

This work was supported by the European Regional Development Fund from the European Union, the “Ministerio de Economía y Competitividad” (Projects AGL2013- 40822-R and AGL2015-65104-P); the “Junta de Andalucía” (Research Groups AGR115 and BIO159) and the economic contribution of the Company TimacAgro Spain (Roullier Group).

## Conflict of Interest

The authors declare that the research was conducted in the absence of any commercial or financial relationships that could be construed as a potential conflict of interest.

## References

[B1] Abel S. (2011). Phosphate sensing in root development. Curr. Opin. Plant Biol. 14, 303–309. 10.1016/j.pbi.2011.04.007 21571579

[B2] Bari R. Pant B. D. Stitt M. Scheible W. R. (2006). PHO2, microRNA399, and PHR1 define a phosphate- pathway in plants. Plant Physiol. 141, 988–999. 10.1104/pp.106.079707 16679424 PMC1489890

[B3] Borch K. Bouma T. J. Lynch J. P. Brown K. M. (1999). Ethylene: a regulator of root architectural responses to soil phosphorus availability. Plant Cell Environ. 22, 425–431. 10.1046/j.1365-3040.1999.00405.x

[B4] Bhosale R. Giri J. Pandey B. K. Giehl R. F. Hartmann A. Traini R. (2018). A mechanistic framework for auxin dependent *Arabidopsis* root hair elongation to low external phosphate. Nat. Commun. 9 (1), 1409–1417. 10.1038/s41467-018-03851-3 29651114 PMC5897496

[B5] Brumbarova T. Bauer P. Ivanov R. (2015). Molecular mechanisms governing *Arabidopsis* iron uptake. Trends Plant Sci. 20, 124–133. 10.1016/j.tplants.2014.11.004 25499025

[B6] Buhtz A. Pieritz J. Springer F. Kehr J. (2010). Phloem small RNAs, nutrient stress responses, and systemic mobility. BMC Plant Biol. 10, 64. 10.1186/1471-2229-10-64 20388194 PMC2923538

[B7] Connolly E. L. Campbell N. H. Grotz N. Prichard C. L. Guerinot M. L. (2003). Overexpression of the FRO2 ferric chelate reductase confers tolerance to growth on low iron and uncovers posttranscriptional control. Plant Physiol. 133, 1102–1110. 10.1104/pp.103.025122 14526117 PMC281606

[B8] Chen W. W. Yang J. L. Qin C. Jin C. W. Mo J. H. Ye T. (2010). Nitric oxide acts downstream of auxin to trigger root ferric-chelate reductase activity in response to iron deficiency in *Arabidopsis thaliana*. Plant Physiol. 154, 810–819. 10.1104/pp.110.161109 20699398 PMC2948983

[B9] Chiou T. J. Lin S. I. (2011). Network in sensing phosphate availability in plants. Annu Rev Plant Biol. 62, 185–206. 10.1146/annurev-arplant-042110-103849 21370979

[B10] de Kock P. C. (1955). Iron nutrition of plants at high pH. Soil Sci. 79, 167–175. 10.1097/00010694-195503000-00001

[B11] Delhaize E. Randall P. J. (1995). Characterization of a phosphate accumulator mutant of *Arabidopsis thaliana*. Plant Physiol. 107, 207–213. 10.1104/pp.107.1.207 12228355 PMC161187

[B12] del Pozo J. C. Allona I. Rubio V. Leyva A. de la Peña A. Aragoncillo C. (1999). A type 5 acid phosphatase gene from *Arabidopsis thaliana* is induced by phosphate starvation and by som other types of phosphate mobilising/oxidative stress conditions. Plant J. 19, 579–589. 10.1046/j.1365-313X.1999.00562.x 10504579

[B13] Franco-Zorrilla J. M. González E. Bustos R. Linhares F. Leyva A. Paz-Ares J. (2004). The transcriptional control of plant responses to phosphate limitation. J. Exp. Bot. 55, 285–293. 10.1093/jxb/erh009 14718495

[B14] García M. J. Lucena C. Romera F. J. Alcántara E. Pérez-Vicente R. (2010). Ethylene and nitric oxide involvement in the up-regulation of key genes related to iron acquisition and homeostasis in *Arabidopsis*. J. Exp. Bot. 61, 3885–3899. 10.1093/jxb/erq203 20627899

[B15] García M. J. Suárez V. Romera F. J. Alcántara E. Pérez-Vicente R. (2011). A new model involving ethylene, nitric oxide and Fe to explain the regulation of Fe-acquisition genes in Strategy I plants. Plant Physiol. Biochem. 49, 537–544. 10.1016/j.plaphy.2011.01.019 21316254

[B16] García M. J. Romera F. J. Stacey M. G. Stacey G. Villar E. Alcántara E. (2013). Shoot to root communication is necessary to control the expression of iron-acquisition genes in Strategy I plants. Planta 237, 65–75. 10.1007/s00425-012-1757-0 22983673

[B17] García M.J. García-Mateo M.J. Lucena C. Romera F.J. Rojas C.L. Alcántara E. (2014). Hypoxia and bicarbonate could block the expression of iron acquisition genes in Strategy I plants by affecting ethylene synthesis and signaling in different ways. Physiol. Plant. 150, 95–106. 10.1111/ppl.12076 23742320

[B18] García M. J. Romera F. J. Lucena C. Alcántara E. Pérez-Vicente R. (2015). Ethylene and the regulation of physiological and morphological responses to nutrient deficiencies. Plant Physiol. 169, 51–60. 10.1104/pp.15.00708 26175512 PMC4577413

[B19] García M. J. Corpas F. J. Lucena C. Alcántara E. Pérez-Vicente R. Zamarreño Á.M. (2018). A shoot Fe pathway requiring the OPT3 transporter controls GSNO reductase and ethylene in *Arabidopsis thaliana* roots. Front. Plant Sci. 9, 1325–1341. 10.3389/fpls.2018.01325 30254659 PMC6142016

[B20] Graziano M. Lamattina L. (2007). Nitric oxide accumulation is required for molecular and physiological responses to iron deficiency in tomato roots. Plant J. 52, 949–960. 10.1111/j.1365-313X.2007.03283.x 17892445

[B21] Ham B. K. Chen J. Yan Y. Lucas W. J. (2018). Insights into plant phosphate sensing and signaling. Curr. Opi. Biotech 49, 1–9. 10.1016/j.copbio.2017.07.005 28732264

[B22] Henry J. B. McCall I. Jackson B. Whipker B. E. (2017). Growth response of herbaceous ornamental to phosphorus fertilization. Hortscience 52, 1362–1367. 10.21273/HORTSCI12256-17

[B23] Hirsch J. Marin E. Floriani M. Chiarenza S. Richaud P. Nussaume L. (2006). Phosphate deficiency promotes modification of iron distribution in *Arabidopsis* plants. Biochimic. 88, 1767–1771. 10.1016/j.biochi.2006.05.007 16757083

[B24] Ivanov R. Brumbarova T. Bauer P. (2012). Fitting into the harsh reality: regulation of iron-deficiency responses in dicotyledonous plants. Mol. Plant 5, 27–42. 10.1093/mp/ssr065 21873619

[B25] Kumar S. Verma S. Trivedi P. K. (2017). Involvement of Small RNAs in phosphorus and sulfur sensing, and stress: current update. Front. Plant. Sci. 8, 285. 10.3389/fpls.2017.00285 28344582 PMC5344913

[B26] Lei M. Zhu C. Liu Y. Karthikeyan A. S. Bressan R. A. Raghothama K. G. (2011). Ethylene is involved in regulation of phosphate starvation-induced gene expression and production of acid phosphatases and anthocyanin in *Arabidopsis*. New Phytol. 189, 1084–1095. 10.1111/j.1469-8137.2010.03555.x 21118263

[B27] Liu T. Y. Huang T. K. Tseng C. Y. Lai Y. S. Lin S. I. Lin W. Y. (2012). PHO2-dependent degradation of PHO1 modulates phosphate homeostasis in *Arabidopsis*. Plant Cell 24 (5), 2168–2183. 10.1105/tpc.112.096636 22634761 PMC3442594

[B28] Liu W. Li Q. Wang Y. Wu T. Yang Y. Zhang X. (2017). Ethylene response factor AtERF72 negatively regulates *Arabidopsis thaliana* response to iron deficiency. Biochem. Biophys. Res. Commun. 491, 862–868. 10.1016/j.bbrc.2017.04.014 28390898

[B29] Lucena C. Waters B. M. Romera F. J. García M. J. Morales M. Alcántara E. (2006). Ethylene could influence ferric reductase, iron transporter and H^+^-ATPase gene expression by affecting FER (or FER-like) gene activity. J. Exp. Bot. 57, 4145–4154. 10.1093/jxb/erl189 17085755

[B30] Lucena C. Romera F. J. Rojas C. L. García M. J. Alcántara E. Pérez-Vicente R. (2007). Bicarbonate blocks the expression of several genes involved in the physiological responses to Fe deficiency of Strategy I plants. Funct. Plant Biol. 34, 1002–1009. 10.1071/FP07136 32689428

[B31] Lucena C. Romera F. J. García M. J. Alcántara A. Pérez-Vicente R. (2015). Ethylene participates in the regulation of Fe deficiency responses in Strategy I plants and in rice. Front. Plant Sci. 6, 1056. 10.3389/fpls.2015.01056 26640474 PMC4661236

[B32] Lucena C. Porras R. Romena F. J. Alcántara E. Pérez-Vicente R. (2018). Similarities and differences in the acquisition of Fe and P by dicot plants. Agronomy 8, 148–163. 10.3390/agronomy8080148

[B33] Marschner H. (1995). Mineral nutrition of higher plants. 2nd ed edition. London: Academic Press.

[B34] Meng Z. B. Chen L. Q. Suo D. Li G. X. Tang C. X. Zheng S. J. (2012). Nitric oxide is the shared signalling molecule in phosphorus- and iron deficiency- induced formation of cluster roots in white lupin (*Lupinus albus*). Ann. Bot. 109, 1055–1064. 10.1093/aob/mcs024 22351487 PMC3336943

[B35] Mehra P. Pandey B. K. Giri J. (2017). Improvement in phosphate acquisition and utilization by a secretory purple acid phosphatase (OsPAP21b) in rice. Plant Biotech. J. 15 (8), 1054–1067. 10.1111/pbi.12699 PMC5506657 28116829

[B36] Misson J. Raghothama K. G. Jain A. Jouhet J. Block M. A. Bligny R. (2005). A genome-wide transcriptional analysis using Arabidopsis thaliana Affymetrix gene chips determined plant responses to phosphate deprivation. Proc. Natl. Acad. Sci. Biol. 102, 11934–11939. 10.1073/pnas.0505266102 PMC1188001 16085708

[B37] Miura K. Lee J. Gong Q. Ma S. Jin J. B. Yoo C. Y. (2011). SIZ1 regulation of phosphate starvation induced root architecture remodeling involves the control of auxin accumulation. Plant Physiol. 155, 1000–1012. 10.1104/pp.110.165191 21156857 PMC3032448

[B38] Mora V. Baigorri R. Bacaicoa E. Zamarreño A. M. García-Mina J. M. (2012). The humic acid-induced changes in the root concentration of nitric oxide, IAA and ethylene do not explain the changes in root architecture caused by humic acid in cucumber. Environ. Exp. Bot. 76, 24–32. 10.1016/j.envexpbot.2011.10.001

[B39] Müller R. Morant M. Jarmer H. Nilsson L. Nielsen T. H. (2007). Genome wide analysis of the *Arabidopsis* leaf transcriptome reveals interaction of phosphate and sugar metabolism. Plant Physiol. 143, 156–171. 10.1104/pp.106.090167 17085508 PMC1761981

[B40] Nagarajan V. K. Smith A. P. (2012). Ethylene´s role in phosphate starvation: more than just a root growth regulator. Plant Cell Physiol. 53 (2), 277–286. 10.1093/pcp/pcr186 22199374

[B41] Neumann G. (2016). The role of ethylene in plant adaptations for phosphate acquisition in soils—A review. Front. Plant Sci. 6, 1224. 10.3389/fpls.2015.01224 26834759 PMC4718997

[B42] Pant B. D. Musialak-Lange M. Nuc P. May P. Buhtz A. Kehr J. (2009). Identification of nutrient-responsive *Arabidopsis* and rapeseed microARNs by comprehensive real-time polymerase chain reaction profiling and small RNA sequencing. Plant Physiol. 150, 1541–1555. 10.1104/pp.109.139139 19465578 PMC2705054

[B43] Perea-García A. García-Molina A. Andres-Colas N. Vera-Sirera F. Perez-Amador M. A. Puig S. (2013). *Arabidopsis* copper transport protein COPT2 participates in the cross talk between iron deficiency responses and low-phosphate. Plant Physiol. 162, 180–194. 10.1104/pp.112.212407 23487432 PMC3641201

[B44] Robinson W. D. Park J. Tran H. T. Del Vecchio H. A. Ying S. Zins J. L. (2012). The secreted purple acid phosphatase isozymes AtPAP12 and AtPAP26 play a pivotal role in extracellular phosphate-scavenging by *Arabidopsis thaliana*. J. Exp. Bot. 63 (18), 6531–6542. 10.1093/jxb/ers309 23125358 PMC3504502

[B45] Romera F. J. Alcantara E. de la Guardia M. D. (1992). Effects of bicarbonate, phosphate and high pH on the reducing capacity of Fe-deficient sunflower and cucumber plants. J. Plant Nutr. 15, 1519–1530. 10.1080/01904169209364418

[B46] Romera F. J. Alcántara E. (1994). Iron-deficiency stress responses in cucumber (*Cucumis sativus* L.) roots. A possible role for ethylene? Plant Physiol. 105, 1133–1138. 10.1104/pp.105.4.1133 12232270 PMC159441

[B47] Roldán M. Dinh P. Leung S. McManus M. T. (2013). Ethylene and the responses of plants to phosphate deficiency. AoB PLANTS 5, plt013. 10.1093/aobpla/plt013

[B48] Rubio V. Linhares F. Solano R. Martín A. C. Iglesias J. Leyva A. (2001). A conversed MYB transcription factor involved in phosphate starvation both in vascular plants and in unicellular algae. Genes Dev. 15, 2122–2133. 10.1101/gad.204401 11511543 PMC312755

[B49] Sánchez-Rodriguez A. R. del Campillo M. C. Torrent J. (2013). Phosphate aggravates iron chlorosis in carbonate–iron oxide systems. Plant Soil 373, 31–42. 10.1007/s11104-013-1785-y

[B50] Stacey M. G. Patel A. McClain W. E. Mathieu M. Remley M. Rogers E. E. (2008). The *Arabidopsis* AtOPT3 protein functions in metal homeostasis and movement of iron to developing seeds. Plant Physiol. 146, 589–601. 10.1104/pp.107.108183 18083798 PMC2245856

[B51] Stetter M. G. Benz M. Ludewig U. (2017). Increased root hair density by loss of WRKY6 in *Arabidopsis thaliana*. Peer J. 5, e2891. 10.7717/peerj.2891 28149680 PMC5267569

[B52] Song L. Liu D. (2015). Ethylene and plant responses to phosphate deficiency. Front. Plant Sci. 6, 796. 10.3389/fpls.2015.00796 26483813 PMC4586416

[B53] Thimm O. Essigmann B. Kloska S. Altmann T. Buckhout T. J. (2001). Response of *Arabidopsis* to iron deficiency stress as revealed by microarray analysis. Plant Physiol. 127 (3), 1030–1043. 10.1104/pp.010191 11706184 PMC129273

[B54] Todd C. D. Zeng P. Rodríguez A. M. Hoyos. M. E. Polacco J. C. (2004). Transcripts of MYB-like genes respond to phosphorus and nitrogen deprivation in *Arabidopsis*. Planta 219, 1003–1009. 10.1007/s00425-004-1305-7 15592750

[B55] Walker E. L. Connolly E. L. (2008). Time to pump iron: iron-deficiency mechanisms of higher plants. Curr. Opin. Plant Biol. 11, 530–535. 10.1016/j.pbi.2008.06.013 18722804

[B56] Wang Y. H. Garvin D. F. Kochian L. V. (2002). Rapid introduction of regulatory and transporter genes in response to phosphorus, potassium, and iron deficiencies in tomato roots. Evidence for cross talk and root/rhizosphere-mediated signals. Plant Physiol. 130, 1370. 10.1104/pp.008854 PMC166655 12428001

[B57] Wang K. L. C. Li H. Ecker J. R. (2002). Ethylene biosynthesis and networks. Plant Cell 2002, S131–S151. 10.1105/tpc.001768 PMC151252 12045274

[B58] Wang X. Wang Y. Tian J. Lim B. L. Yan X. Liao H. (2009). Overexpressing AtPAP15 enhances phosphorus efficiency in soybean. Plant Physiol. 151, 233–240. 10.1104/pp.109.138891 19587103 PMC2736008

[B59] Wang Z. Straub D. Yang H. Kania A. Shen J. Ludewig U. (2014). The regulatory network of cluster-root function and development in phosphate deficient white lupin (*Lupinus albus*) identified by transcriptome sequencing. Physiol. Plant 151, 323–338. 10.1111/ppl.12187 24635386

[B60] Wang F. Deng M. Xu J. Zhu X. Mao C. (2018). Molecular mechanisms of phosphate transport and in higher plants. Semin. Cell Develop. Biol. 74, 114–122. 10.1016/j.semcdb.2017.06.013 28648582

[B61] Wang L. Liu D. (2018). Functions and regulation of phosphate starvation-induced secreted acid phosphatases in higher plants. Plant Sci. 271, 108–116. 10.1016/j.plantsci.2018.03.013 29650148

[B62] Ward J. T. Lahner B. Yakubova E. Salt D. E. Raghothama K. G. (2008). The effect of iron on the primary root elongation of *Arabidopsis* during phosphate deficiency. Plant Physiol. 147, 1181–1191. 10.1104/pp.108.118562 18467463 PMC2442553

[B63] Yuan Y. Wu H. Wang N. Li J. Zhao W. Du J. (2008). FIT interacts with AtbHLH38 and AtbHLH39 in regulating iron uptake gene expression for iron homeostasis in *Arabidopsis*. Cell Res. 18, 385–397. 10.1038/cr.2008.26 18268542

[B64] Zakhleniuk O. V. Raines C. A. Lloyd J. C. (2001). Pho3: a phosphorus-deficient mutant of *Arabidopsis thaliana* (L.) Heynh. Planta 212, 529–534. 10.1007/s004250000450 11525509

[B65] Zhai Z. Gayomba S. R. Jung H. Vimalakumari N. K. Piñeros M. Craft E. (2014). OPT3 is a Phloem-specific iron transporter that is essential for systemic iron and redistriburtion of iron and cadmium in *Arabidopsis*. Plant Cell 26, 2249–2264. 10.1105/tpc.114.123737 24867923 PMC4079381

[B66] Zhang Z. Hong L. William J. L. (2014). Molecular mechanisms underlying phosphate sensing, and adaptation in plants. J. Integrative Plant Biol. 56, 192–220. 10.1111/jipb.12163 24417933

[B67] Zheng L. Huang F. Narsai R. Wu J. Giraud E. He F. (2009). Physiological and transcriptome analysis of iron and phosphorus interaction in rice seedlings. Plant Physiol. 151, 262–274. 10.1104/pp.109.141051 19605549 PMC2735995

